# Hepatic functional and pathological changes of type 1 diabetic mice in growing and maturation time

**DOI:** 10.1111/jcmm.14504

**Published:** 2019-06-20

**Authors:** Saizhi Jiang, Xiaoqiang Tang, Kai Wang, Yaqing Liang, Yan Qian, Chaosheng Lu, Lu Cai

**Affiliations:** ^1^ Department of Pediatrics, The First Affiliated Hospital of Wenzhou Medical University Wenzhou Medical University Wenzhou China; ^2^ Department of Pediatrics, Pediatric Research Institute University of Louisville School of Medicine Louisville Kentucky; ^3^ The Center of Cardiovascular Disorders The First Hospital of Jilin University Changchun China; ^4^ Department of Pharmacology and Toxicology University of Louisville School of Medicine Louisville Kentucky

**Keywords:** early life, gender, lipids, liver, type 1 diabetes

## Abstract

To detect the changes in the liver function in both male and female OVE26 mice from young to adults for better understanding of type 1 diabetes‐induced hepatic changes, OVE26 mice and wild‐type FVB mice were raised in the same environment without any intervention, and then killed at 4, 12, 24 and 36 weeks for examining liver's general properties, including pathogenic and molecular changes. The influence of diabetes on the bodyweight of male and female mice was different. Both male and female OVE26 mice did not obtain serious liver injury or non‐alcoholic fatty liver disease, manifested by mild elevation of plasma alanine transaminase, and less liver lipid content along with significantly suppressed lipid synthesis. Uncontrolled diabetes also did not cause hepatic glycogen accumulation in OVE26 mice after 4 weeks. Oxidative stress test showed no change in lipid peroxidation, but increased protein oxidation. Changed endoplasmic reticulum stress and apoptosis along with increased antioxidant capacity was observed in OVE26 mice. In conclusion, uncontrolled type 1 diabetes did not cause hepatic lipid deposition most likely because of reduced lipids synthesis in response to insulin deficiency. Enhanced antioxidant capacity might not only prevent the occurrence of severe acute liver injury but also the self‐renewal, leading to liver dysfunction.

## 1 INTRODUCTION

Diabetes is a widespread disease, which is a serious threat to human health. Type 2 diabetes (T2D) is the most common type of diabetes, however, most childhood diabetes mellitus is type 1 diabetes (T1D). Because of the early onset of T1D in life, the influence of diabetes on the development of important organs is worthy of research.

The liver is a vitally important metabolic organ that regulates glucose and lipid metabolism.^1^, ^2^ Liver diseases in diabetic patients have been extensively researched and described earlier, but mainly in T2D cases^3^, ^4^ whereas few have been conducted in T1D cases, especially in cases of childhood onset. Therefore, it is meaningful to examine in deeper details the liver damage caused in congenital mice model.

OVE26 mice was previously generated by introduction of a calmodulin transgene regulated by an insulin promoter into FVB mice, which caused beta cell‐specific damage,^5^ without the effects of streptozotocin (STZ) toxicity. Such mice spontaneously develop T1D in less than seven days after birth, eventually leading to severe hyperglycaemia^5^, ^6^ with an elevated plasma TG level, which is considered as a manifestation of poorly controlled T1D.^7^ Previous studies have shown obvious cardiovascular^8^, ^9^, ^10^ and renal damage in OVE26 mice; these mice exhibited the distinct albuminuria, characteristic for human T1D and diabetic nephropathy.^6^, ^11^

In our previous work, OVE26 male mice showed hepatic injury,^9^, ^12^ but there's a lack of dynamic examination and female OVE26 mice data. We hypothesized that the liver damage in T1D mice may be time‐dependent and have gender difference since many studies showed the female hormone's protection from various pathogenesis. Therefore, in order to study the effect of T1D on liver function during developing and maturation stage, we designed the experiment involving male and female mice from pups to adults.

## 2 MATERIALS AND METHODS

### 2.1 Animals

Diabetic (OVE26) mice and age, gender‐matched wild‐type (WT, FVB) mice were housed at the University of Louisville Research Resources Center with a 12‐h light/dark cycle at 22°C and fed on standard pellet chow and water ad libitum. OVE26 mice and FVB mice were raised in the same environment without any intervention for whole life. Mice were killed without fasting between 10:00 am and 5:00 pm after a vertin‐anaesthesia. All animal procedures were approved by the Institutional Animal Care and Use Committee of the University of Louisville, and were performed in accordance with the Guide for the Care and Use of Laboratory Animals published by the US National Institutes of Health (NIH Publication No. 85‐23, revised 1996).

According to gender and strain, four different mice including FVB male mice (FM), FVB female mice (FF), OVE male mice (OM) and OVE female mice (OF) were killed at 4 age stages (4, 12, 24, and 36 weeks), equivalent to early puberty, early adulthood, middle age in human. The number of mice in each group is more than 5 (4FM: n = 11, 4FF: n = 11, 4OM: n = 11, 4OF: n = 11, 12FM: n = 10, 12FF: n = 11, 12OM: n = 8, 12OF: n = 9, 24FM: n = 10, 24FF: n = 11, 24OM: n = 6, 24OF: n = 8, 36FM: n = 7, 36FF: n = 10, 36OM: n = 8, 36OF: n = 9).

### 2.2 Western blot analysis

Western blotting was done as previously described.^13^, ^14^ Antibodies are listed in Table S1.

### 2.3 RNA isolation and real‐time RT‐PCR

Total RNA was extracted from liver tissues using TRIzol reagent (RNA STAT 60 Tel‐Test; Ambion, Austin, TX, USA). RNA concentrations were determined spectrophotometrically, genomic DNA was eliminated from the extracted RNA using RapidOut DNA Removal Kit (Thermo Fisher Scientific, Grand Island, NY, USA, K2981). The concentration was re‐determined after purification, and then 1 μg of RNA was reversely transcribed using an avian myeloblastosis virus reverse‐transcriptase kit (Promega, Madison, WI, USA). TaqMan Universal PCR Master Mix (Applied Biosystems) was employed to prepare the PCR mix; the primers utilized are listed in Table S2. The amplification reactions were realized in a Light Cycler ® 96 Detection System (Roche Applied Science, Mannheim, Germany) with initial hold steps (95°C for 10 minutes) and 45 cycles of a two‐step PCR (95°C for 10 seconds and 60°C for 45 seconds). The fluorescence intensity of each sample was measured at each temperature change to monitor the amplification of the target gene.

### 2.4 Determination of lipid peroxidation

Liver lipid peroxidation was examined using thiobarbituric acid–reactive substances assay according to the formation of malondialdehyde (MDA) during acid hydrolysis of the lipid peroxide compound, as previously described.^15^

### 2.5 Haematoxylin‐and‐eosin staining and Oil Red O staining

Hepatic tissues were fixed in 10% neutral phosphate‐buffered formalin. Tissues were embedded in paraffin and sectioned to a thickness of 5 µm for histopathological examination. Haematoxylin and eosin (H&E)‐stained sections were microscopically evaluated. Oil Red O (ORO) staining of the lipid accumulation in the liver tissues was performed in optimal cutting temperature medium (OCT)‐embedded frozen tissue. Cryosections (10‐mm thick) from the OCT‐embedded liver tissues were fixed in 4% buffered formalin for 10 minutes at room temperature, and stained with Oil Red O dye for 30 minutes.^16^ All images were measured at 20x magnification and analysed using ImageJ version 1.44(https://imagej.nih.gov/ij/) software.

### 2.6 Biochemical analysis

To analyse the liver injury and metabolic abnormalities in the liver, the levels of plasma alanine aminotransferase (ALT), plasma glucose, plasma insulin, plasma and liver triglyceride (TG), and liver cholesterol (TC) were determined. The plasma ALT was measured using an ALT Colorimetric Activity Assay kit (Cayman Chemical Company, Ann Arbor, MI, USA), according to the instructions of the manufacturer provided. Plasma insulin was measured using an Ultra Sensitive Mouse Insulin ELISA Kit, 90080 (Crystal Chem Inc, Downers Grove, IL, USA), according to the instruction provided. Plasma glucose assay was performed using a Mouse Glucose Assay Kit, 81692 (Crystal Chem Inc, Downers Grove, IL, USA). TG assay was conducted using a TG infinity assay kit (Thermo Fisher Scientific Inc, Waltham, MA, USA). Additionally, a TC assay was carried out using the TC infinity assay kit (Thermo Fisher Scientific Inc, Waltham, MA, USA).

### 2.7 Immunofluorescence analysis

To assess the changes in the hepatocyte size, frozen tissue was cut into 8‐μm sections. Then, the sections were washed with PBS and stained with FITC‐conjugated phalloidin (Alexa Fluor‐488; Invitrogen, Carlsbad, CA, USA) as previously described.^9^ Fluorescence was measure at 585‐nm and analysed using ImageJ.

### 2.8 Glycogen determination

Glycogen was measured as previously described.^17^

### 2.9 Statistical analyses

Data are presented as mean ± SD. Statistical analyses were conducted using the *t* test or one‐ or two‐way ANOVA as specified in each figure legend (Prism version 5; GraphPad Software, San Diego, CA, USA). A *P* < 0.05 was considered statistically significant.

## 3 RESULTS

### 3.1 General performance and liver pathogenic alterations of OVE26 mice

**Figure 1:**
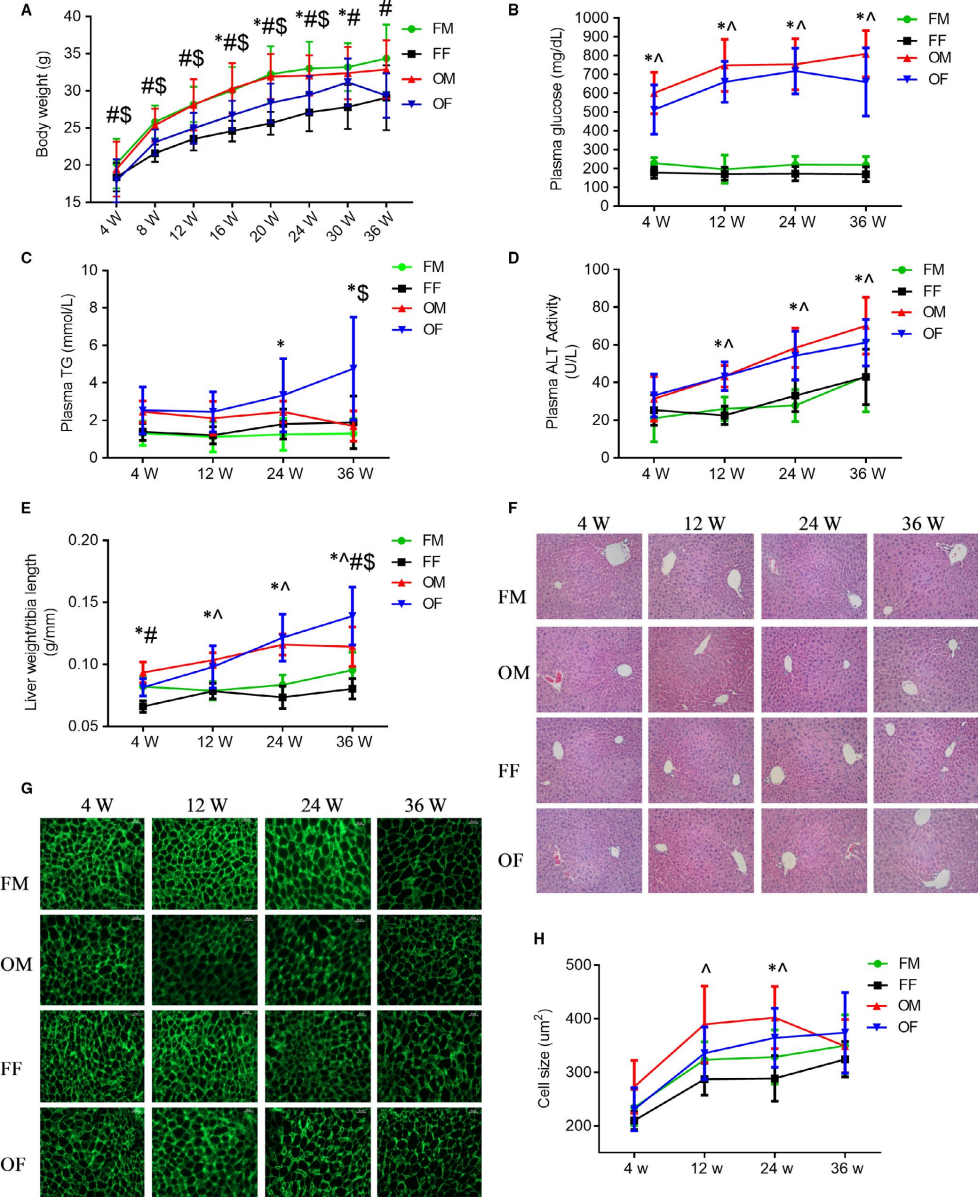
General features of OVE26 mice plasma and liver. A, Bodyweight. B, Plasma glucose. C, Plasma TG change. D, Plasma ALT levels. E, Liver weight, as liver weight to tibia length ratio. F, Liver tissue H&E staining (scale bar = 100 um). G, FITC‐conjugated phalloidin staining of liver sections (scale bar = 25 um). H, Quantification of hepatocyte size between FVB and OVE26 mice. Data were compared by two‐way ANOVA followed by Tukey's multiple comparisons test (*P < 0.05 FF vs OF, ^*P* < 0.05 FM vs OM, #*P* < 0.05 FM vs FF, $*P* < 0.05 OM vs OF). Data are presented as the mean ± SD

Body‐weight in both male and female FVB mice increased in age‐dependent manner from 4 to 20 weeks of age, and females continue to gain body‐weight after 20 weeks old as compare to males, but males were significantly heavier than females all the time. Compared to the bodyweight of gender‐ and age‐matched FVB mice, the bodyweight of male OVE26 mice was not significantly changed, but was increased in female OVE26 mice at ages between 16‐30 weeks. OVE26 mice also showed age‐dependent body‐weight increase in both male and female, except for female OVE26 mice at 36 weeks, and males were significantly heavier than females from 4 to 24 weeks of age (*P* < 0.05) (Figure 1A). Specific bodyweight changes were supplied in Figure S1.

No difference for the plasma glucose levels between male and female FVB mice, but compared to FVB both male and female OVE26 mice showed significantly increased plasma glucose levels at approximately threefold at 4 weeks old to fourfold at 12 weeks old and forward. There was no significant gender difference among OVE26 mice for plasma glucose level (Figure 1B).

Plasma TG assay revealed a slight trend of age‐dependent increase after 12 weeks in female FVB mice compared to same aged FVB males (*P* > 0.05). Female OVE26 mice showed significantly increased level of plasma TG in an age‐dependent manner after 12 weeks (*P* < 0.05 vs gender‐ and age‐matched FVB). Between OVE26 males and females, there was a statistically significant difference only at 36 weeks old (Figure 1C).

As an index of hepatic injury, plasma ALT displayed a stable level (about 20 U/L) within 4–12 weeks old in both male and female FVB mice. For OVE26 mice, both male and female mice showed statistically increased ALT level compared to FVB from 12‐36 weeks in an age‐dependent manner without gender difference although OVE26 females showed a little bit lower (*P* > 0.05) than OVE26 males at the 36 weeks old (Figure 1D).

**Figure 2:**
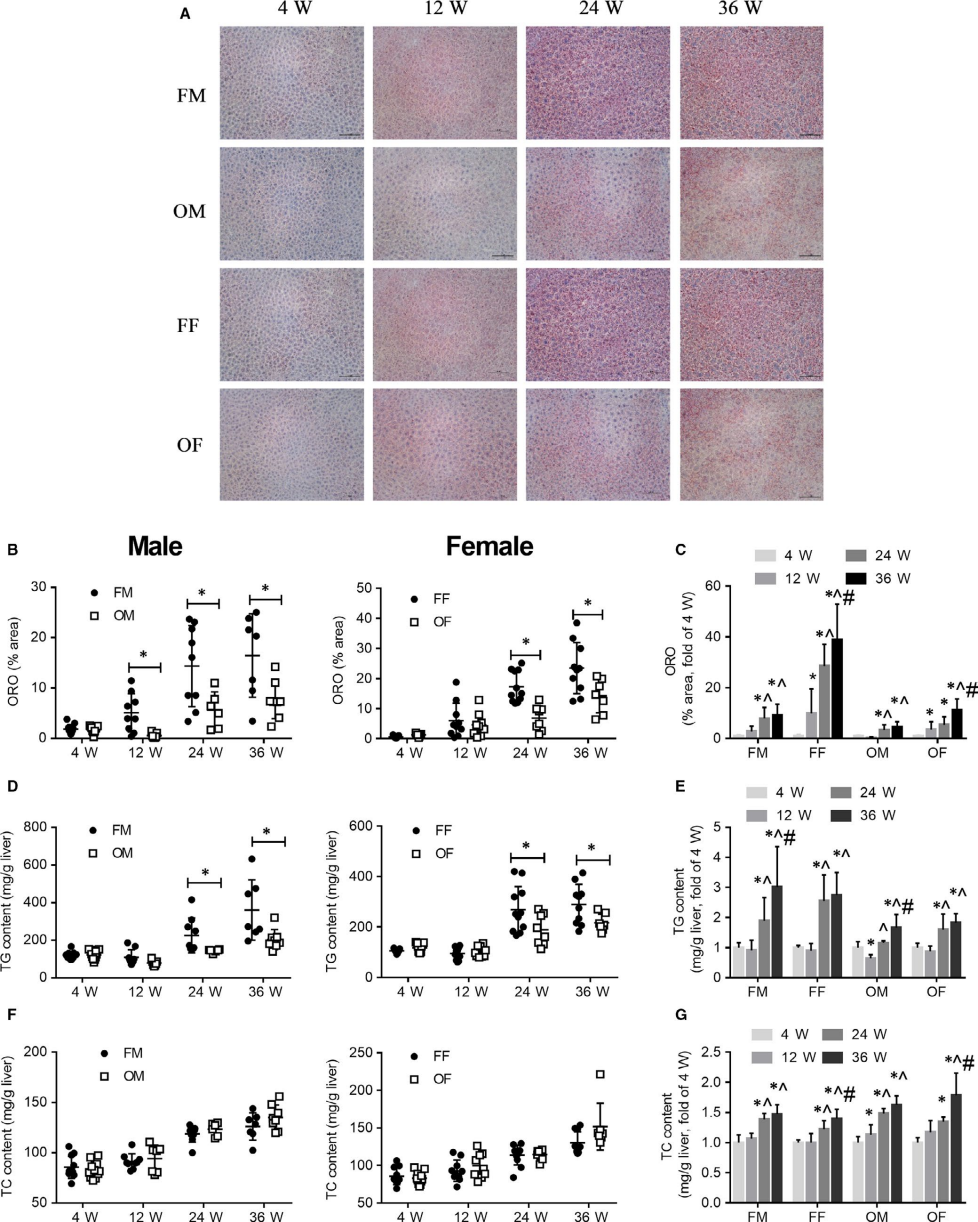
OVE mice showed lower lipid content in the liver than FVB mice with ageing; both FVB and OVE mice had an increase in hepatic lipids with age. A, Oil red O staining (scale bar = 100 um). B, Quantification of red lipid areas between FVB and OVE26 mice. C, Quantification of red lipid areas in age. D, Liver tissue TG change between FVB and OVE26 mice. E, Liver tissue TG change with ageing. F, Liver tissue TC change between FVB and OVE26 mice. G, Liver tissue TC change in age. In B, D and F, Data were compared by unpaired *t* test, *P < 0.05 vs FVB mice of the same age and gender. In C, E and G, Data were compared by one‐way ANOVA followed by Tukey test, *P < 0.05 vs 4 weeks old; ^*P* < 0.05 vs 12 weeks old; #*P* < 0.05 vs 24 weeks old. Data were presented as means ± SD

The general ratios of liver weight to tibia length, used as an index of the liver size changes, were slightly higher in the FVB males than in the FVB females (*P* < 0.05) only at ages of 4 and 36 weeks. Compared to age‐matched FVB mice, OVE26 female mice showed more pronounced hepatomegaly at all ages, while OVE26 male mice at 12, 24 and 36 weeks (*P* < 0.05) (Figure 2A). Concerning sexual difference, female OVE26 mice showed a lower ratio of the liver to tibia length compared to the males at 4 weeks of age(*P* > 0.05), but it increased faster forward and was significantly larger at 36 weeks (Figure 1E).

HE staining showed no obvious pathological change in OVE26 mice liver (Figure 1F). To explore hepatomegaly showed in Figure 1E, we performed phalloidin staining of actin filaments, so that we can measure hepatic cell sizes (Figure 1G). All the mice showed time‐dependent increase, hepatocyte size became bigger after the age of 4 weeks, except for the male OVE26 mice that showed quickly decreased size from 24 to 36 weeks. Male mice showed larger hepatic cells compared to females (*P* > 0.05), except for male OVE26 mice at 36 weeks. Compared to FVB, OVE26 male mice showed significant bigger hepatocytes than FVB mice at 12 and 24 weeks, while OVE26 female mice only at 24 weeks (Figure 1H).

### 3.2 Type 1 Diabetes did not induce lipids deposition in OVE26 mice liver due to reduced lipids synthesis. Hepatic glycogen accumulation was not found in OVE26 mice after 4 weeks old

Diabetes is a disease in which glucose and lipid metabolism is dysregulated, next we detected lipids in liver. ORO staining revealed the availability of fewer neutral lipids in the liver of male OVE26 mice after 4 weeks of age as well as in the liver of female OVE26 mice after an age of 12 weeks (Figure 2A,B). We found an increase in the liver neutral lipids content with ageing both in FVB and OVE26 mice; in male mice that change started from 24 weeks of age and in female mice the change started from 12 weeks old (Figure 2C). Further biochemical analysis‐TG and TC test confirmed the age‐related increase in lipids (Figure 2E and G). The TG test also showed a reduced TG content in the liver of OVE26 mice after an age of 12 weeks (Figure 2D). Nevertheless, the TC test results did not exhibit differences in this property between FVB and OVE26 mice (Figure 2F).

**Figure 3:**
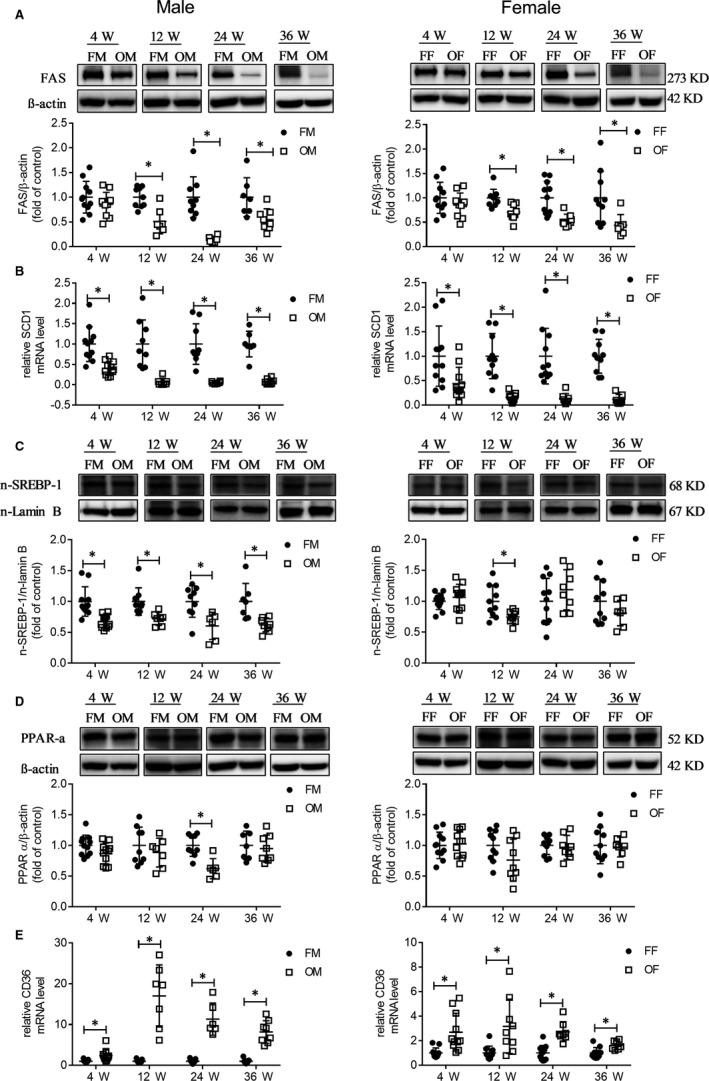
Reduced lipids synthesis was the cause of less lipids in OVE mice liver. A, FAS protein expression between FVB and OVE26 was detected by Western blot. B, mRNA expression of SCD1 between FVB and OVE26. C, SREBP‐1 nuclear translocation was determined by Western blot. D, PPAR‐α protein expression between FVB and OVE26 was detected by Western blot. E, mRNA expression of CD36 between FVB and OVE26. Data were compared by unpaired *t* test, *P < 0.05 vs FVB mice of the same age and gender. Data are expressed as means ± SD

To identify the possible causes of lipids reduction in OVE26 mice, FAS (lipid synthesis), PPAR‐α (lipid oxidation), LC3B and P62 (autophagy) were determined by Western blot analyses. SCD1 (lipid synthesis) and CD36 (fatty acid uptake) expression were measured by a real‐time PCR assay. FAS and SCD1 test results revealed that the lipid synthesis was reduced in both male and female OVE26 mice from an early age (Figure 3A,B). It is known that SREBP‐1 regulates the expression of the genes, required for fatty acid and lipid production, through regulation of insulin levels,^18^ male OVE26 mice showed reduced nuclear translocation of SREBP‐1, and the trend was consistent with the trend of changes in FAS and SCD1. However, female OVE26 mice showed only reduced nuclear translocation of SREBP‐1 at an age of 12 weeks, indicating that a different regulation mechanism operated between male and female animals (Figure 3C).

**Figure 4:**
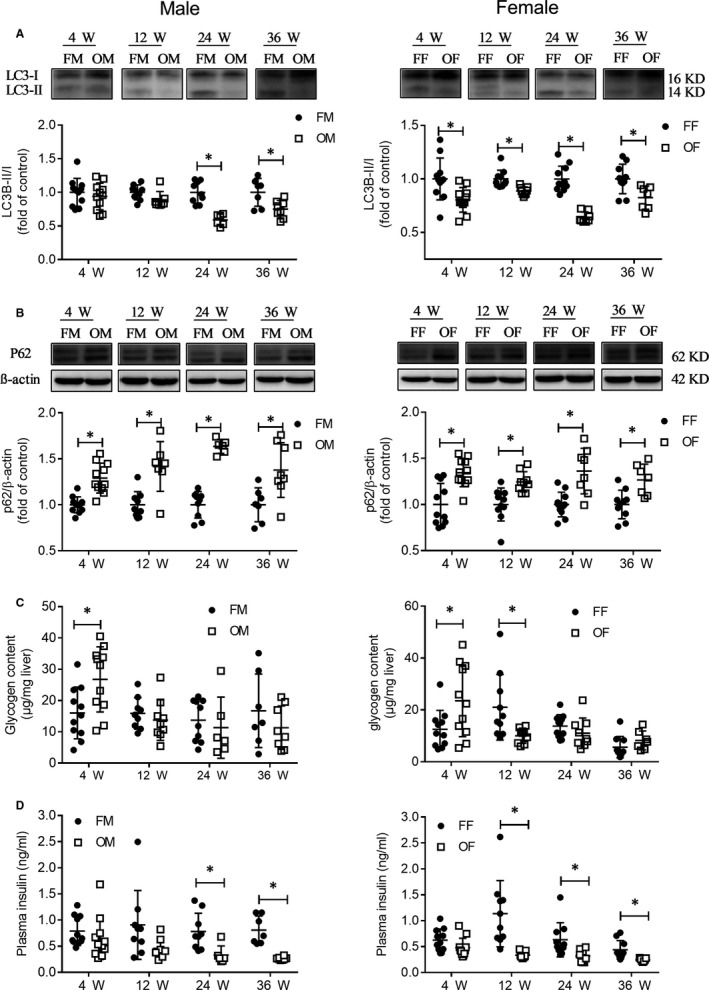
Changes of liver autophagy protein, hepatic glycogen content and plasma insulin level. A, LC3B protein expression was detected by Western blot. B, P62 protein expression was detected by Western blot. C, Liver tissue glycogen content. D, Plasma insulin levels. Data were compared by unpaired *t* test, * *P* < 0.05 vs FVB mice in same age and gender. Data were presented as means ± SD

PPAR‐α result showed no increase in the lipid oxidation in the liver of OVE26 mice at most ages studied, but reduced lipid oxidation at 24 weeks old was found in diabetic male mice (Figure 3D). The increased CD36 expression indicated the presence of an augmented ability of OVE26 mice to uptake fatty acids (Figure 3E). OVE26 mice exhibited reduced autophagy as shown in LC3B (Figure 4A) and P62 (Figure 4B), and the trend in the female mice was more obvious than in the male.

Next, we assessed glycogen content in liver tissue. The results showed that more glycogen content was only found at 4 weeks old in OVE26 mice liver both male and female. After 4 weeks old, there was no difference between OVE26 male and FVB male mice. For female mice, we found increased glycogen content in FVB mice at 12 weeks old, after that there was no difference between OVE26 and FVB mice (Figure 4C). Plasma insulin levels were assessed (Figure 4D). For male mice, plasma insulin levels in OVE26 mice significantly reduced after 12 weeks old. For female mice, plasma insulin levels in OVE26 mice significantly reduced after 4 weeks old. Still a low level of insulin exists in OVE26 mice, the results are similar to previous reports.^5^, ^6^, ^19^

### 3.3 Oxidative stress, inflammation and endoplasmic reticulum (ER) stress

**Figure 5:**
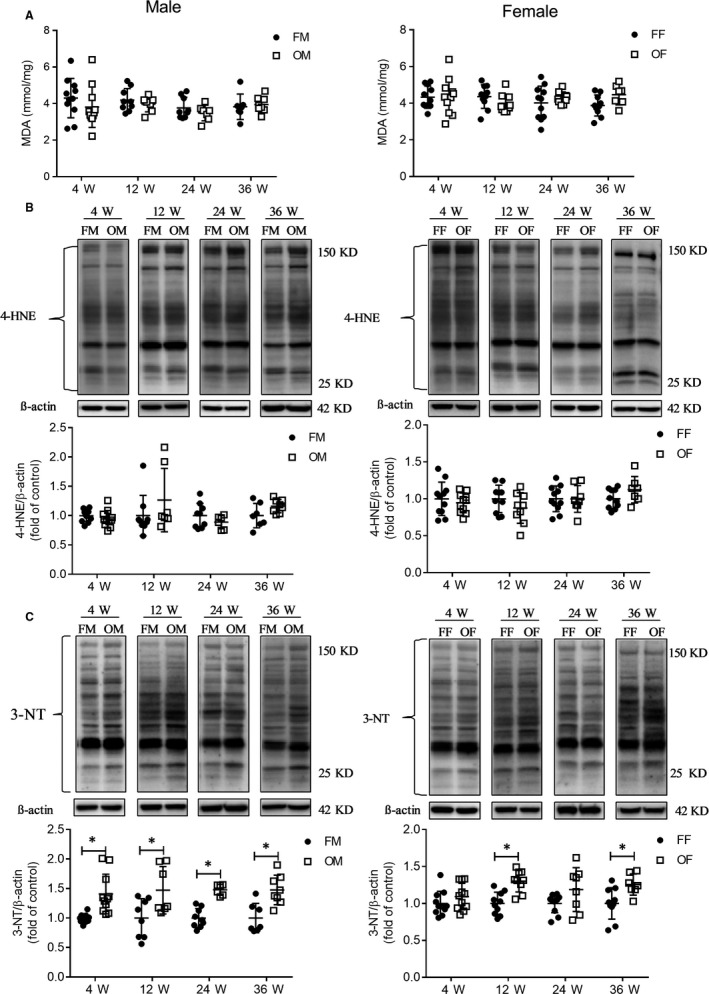
Oxidative stress test showed that protein peroxidation occurred in OVE26 mice liver, and lipid peroxidative damage did not occur in the OVE mice liver. A, Lipid peroxidation (MDA) in the liver. B, 4‐HNE protein expression was detected by Western blot. C, Protein peroxidation revealed by 3‐NT protein levels detected by Western blot. Data were compared using unpaired *t* test, *P < 0.05 vs FVB mice in same age and gender. Data were presented as means ± SD

Oxidative stress damage is the most widely recognized way through which diabetes induces organ damage. Hence, to confirm the above results, we additionally examined the levels of lipid peroxidation and protein oxidation. The MDA assay (Figure 5A) and western‐blot of 4‐HNE (Figure 5B) results showed no increase in the lipid oxidation in OVE26 mice liver in both male and female animals.

As can be seen in Figures 5C, 3‐NT results revealed the presence of multiple nitrated proteins, induced by diabetes. We detected higher nitration levels of proteins in OVE26 male mice at all four ages, but only at 12 weeks and 36 weeks of age in OVE26 female mice. It is noteworthy that less severe oxidative damage was observed in the OVE26 female mice than OVE26 male mice.

**Figure 6:**
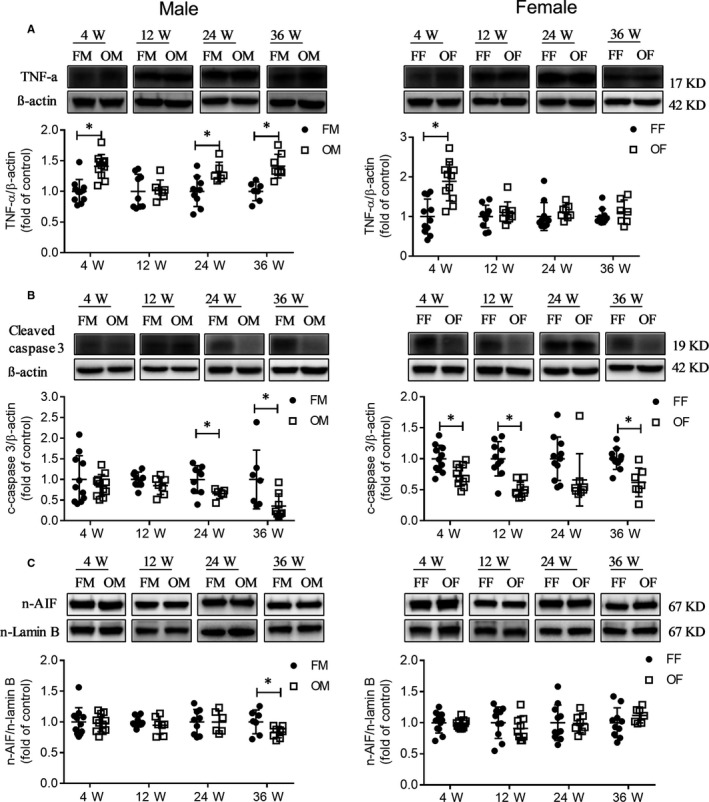
Liver inflammation and apoptosis in OVE26 mice. A, TNF‐α protein expression was detected by Western blot. B, cleaved caspase‐3 protein expression was detected by Western blot. C, AIF nuclear translocation was detected by Western blot. Data were compared by unpaired *t* test, *P < 0.05 vs FVB mice in same age and gender. Data were presented as means ± SD

Since protein nitration in OVE26 mice were found, next we detected whether T1D induce inflammation and cell death in the livers of OVE26 mice. Except for the age of 12 weeks, in the other three age groups, hepatic TNF‐α level in OVE26 male mice increased, whereas increased TNF‐α levels were found only in female OVE 26 mice at 4 weeks of age (Figure 6A), the results suggested that age and gender exert an impact on the inflammatory response in severe T1D.

Western blot results showed that OVE26 mice had less apoptosis compared to FVB mice as revealed by the expression of cleaved caspase‐3 (Figure 6B) and AIF nuclear translocation (Figure 6C). Male and female mice displayed different patterns of changes in their ER stress at different ages. For example, the nuclear translocation of ATF6 and CHOP expression in OVE26 male mice was reduced at most ages, whereas an increase in this parameter in OVE26 female mice was present at an age of 4 weeks and no change was detected at other ages (Figure S2A and B). We also detected the BiP expression and nuclear translocation of ATF4 and found that male and female mice had an identical pattern of changes. BiP expression increased at the early ages (4 and 12 weeks) (Figure S2C), but there was no difference in the nuclear transport of ATF4 between FVB and OVE mice (Figure S2D).

### 3.4 Hepatic antioxidative capacity of OVE26 mice

**Figure 7:**
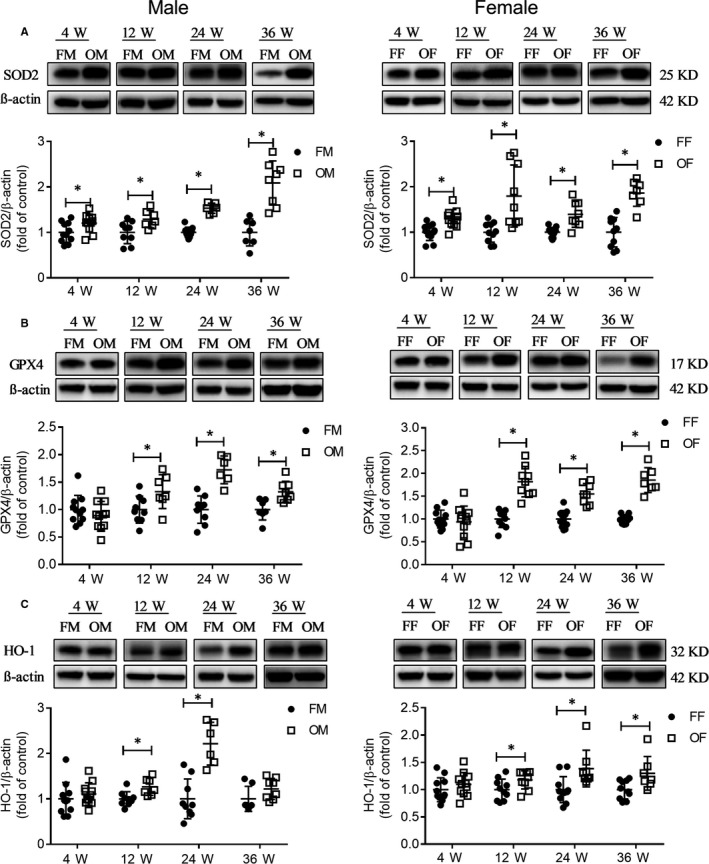
OVE mice showed increased antioxidative ability. A, SOD2 protein expression was detected by Western blot. B, GPX4 protein expression was detected by Western blot. C, HO‐1 protein expression was detected by Western blot. Data were compared by unpaired *t* test, *P < 0.05 vs FVB mice in same age and gender. Data were presented as means ± SD

We found increased protein peroxidation and inflammation but reduced apoptosis and mild general liver injury; these results imply the existence of possible protection mechanisms. As the ability to resist oxidative damage is critical for survival, we next assessed the hepatic antioxidative ability of OVE26 mice. We found that SOD2 had higher expression levels in the liver of OVE26 mice in 4‐36 weeks old (Figure 7A), whereas GPX4 was overexpressed in the liver of OVE26 mice within 12‐36 weeks (Figure 7B). Of note, HO‐1 also had higher hepatic expression in OVE26 mice, but in OVE26 male mice that phenomenon was established only at the ages of 12 and 24 weeks; differently, in OVE26 female mice this change occurred within 12‐36 weeks of age (Figure 7C). These results suggested that OVE26 mice had higher antioxidant capacity than FVB.

## 4 DISCUSSION

To the best of our knowledge, this is the first report on the liver function of OVE26 mice of both sexes from young to adult. (a) In the present study, we found that both male and female OVE26 mice did not exhibit serious acute liver injury especially at young age. (b) After 20 weeks of age, the weight gain of male and female mice was inconsistent, and the influence of diabetes on the weight gain of male and female mice was also different. (c) Lipid analysis results showed that the lipid content in the liver of OVE26 mice was lower than that of FVB mice after 4 weeks old. (d) Glycogen analysis results showed that T1D did not cause hepatic glycogen accumulation in OVE26 mice after 4 weeks old. (e) The performance of male and female OVE26 mice at different age stages were different, and the damage in female mice was less serious than that in the male mice, like other studies found that females generally have lower levels of oxidative stress and ROS production than males in cardiovascular system.^20^ (f) We further found changed apoptosis and autophagy protein levels and an increased antioxidant capacity in OVE26 mice.

Weight gain is common among ageing women, especially during the menopausal transition.^21^ The underlying mechanisms are largely unknown. Pre‐clinical and clinical research indicate that ovarian hormones may play a major role, oestrogens play a leading role in the causes and consequences of female obesity.^22^ After 20 weeks is equivalent to reaching menopause, we hypothesized that oestrogen was responsible for the continued weight gain of female mice. Diabetes affects ovarian function throughout the lifespan, female with T1D are more likely to have menstrual dysfunction and an earlier menopause.^23^ Hypogonadotropic hypogonadism, hypoestrogenism, excessive weight and fat mass gain have been described in T1D women.^24^ The weight gain of OVE26 female mice between 16‐30 weeks was the result of T1D.

Non‐alcoholic fatty liver disease (NAFLD) is the most common liver disease, it is prevalent in T2D and can occur in T1D.^25^ However, the diagnosis of NAFLD has been mainly based on ultrasonography findings, which is not considered the optimal method to measure fatty liver.^26^ In children and adolescents with T1D, screening using ultrasound did not find significantly increased prevalence of NAFLD.^27^ Studies by magnetic resonance (MR) imaging, conducted to measure the hepatic fat content, found no increased prevalence of NAFLD in adults patients with T1D^28^, ^29^, ^30^ and in children patients with T1D.^31^ Insulin is essentially involved in the regulation of the hepatic energy metabolism and promotes lipogenesis in the liver.^32^ In T2D cases defined as insulin‐resistant states, insulin failed to suppress hepatic glucose production but enhanced lipid synthesis.^33^ Lipid synthesis was found to be reduced in T1D, which is characterized by diminished production of insulin.^34^, ^35^ In addition, insulin therapy was established to stimulate the lipogenesis^31^ and weight gain in patients with T1D.^36^ In this study, we found a decrease in FAS expression in both male and female OVE26 mice at the same age, which is in agreement with the findings of the above‐mentioned human studies.

Lipids may accumulate in the liver as a result of multiple abnormalities in the hepatic lipid metabolism, including enhanced fat uptake and lipid synthesis, suppressed lipolysis, lipid oxidation^37^ and inhibited autophagy. The level of PPAR‐α expression, involved in the transcriptional control of genes encoding mitochondrial fatty acid beta‐oxidation enzymes,^38^ was not increased in the liver of OVE26 mice. Fatty acid translocase CD36 mediates the uptake and intracellular transport of long‐chain fatty acids in diverse cell types. In a previous examination, hepatic CD36 up‐regulation was found to be significantly associated with increased fatty liver occurrence.^39^, ^40^ In our investigation, CD36 expression obviously increased in the liver of OVE26 mice. Based on these results, decreased lipid levels were only consistent with reduced lipid synthesis, indicating that the reduced lipids were to be attributed to suppressed lipid synthesis.

Glycogenosis is the hepatic response to excess circulating insulin and glucose in children, adolescence and adults with T1D.^41^ Under unusual T1D conditions, when blood glucose is high and administered insulin is elevated, hepatic glycogenosis can occur resulting in excessive glycogen storage in the liver. It was reported that poorly controlled T1D patients display a marked reduction in hepatic glycogen synthesis, in the absence of elevated insulin level.^42^

Insulin is considered to be the main anabolic hormone of the body, it regulates the metabolism of glucose and fats, and its secretion is regulated by diet and hormones. We found big variations in insulin level of FVB mice especially at 12 weeks old. The secretion of insulin is affected by diet, blood samples in the study for assays were obtained from fed mice, it may be one of the reasons for big variation in FVB mice. 12 weeks old is equivalent to late stage of puberty in mice, previous cross‐sectional studies show that puberty is associated with a reduction in insulin sensitivity, the fall in insulin sensitivity during puberty is associated with a compensatory increase in insulin secretion.^43^ Endocrine changes in puberty may be the reason for big variation at 12 weeks old.

The prevalence of liver disease among T1D is about 20%‐31%^44^, ^45^, ^46^ and related to poor glycaemic control, mainly presenting as increased liver enzymes and hepatomegaly. Harman DJ *et al* found that T1D was associated with a previously unrecognized burden of chronic liver disease.^47^

Autophagy is a cellular self‐protection function, it is a fundamental cellular homeostatic mechanism,^48^ can contribute to cell damage but may also serve to protect cells.^49^ In a mice model with hepatic autophagy deficiency, the protein aggregates and subcellular organelles were massively accumulated, leading to hepatomegaly, severe liver injury, inflammation and fibrosis.^50^ In critically ill patients, because of insufficient activation of autophagy, cellular damage accumulated in organ.^51^ Insufficiently activated autophagy in long‐term illness has shown to associate with organ failure.^52^ Autophagy was essentially involved in lipid metabolism regulation, the inhibition of autophagy increased the in vivo and in vitro storage of triglycerides in lipid droplets.^53^ The present study suggests that autophagy may be reduced in OVE26 mice liver. We assumed that reduced autophagy in OVE26 mice liver lead to clearance disorders for misfolded proteins and abnormal organelles, resulting in hepatomegaly and dysfunction, not likely due to excess glycogen or fat in the liver.^44^

Apoptosis is a naturally occurring cell death process, essential for the normal development and homeostasis of all multicellular organisms.^54^ This process is also important for removing damaged, infected or potentially neoplastic cells.^55^ However, abnormal levels in both directions, extremely low or high apoptotic cell death levels, can lead to adverse biological consequences such as acute liver failure or hepatocellular carcinoma.^56^ Cell death is at the centre of acute and chronic liver disease.^57^ Whether decreased liver apoptosis in OVE26 mice will protect the liver from diabetes damage or hinder the self‐renewal of the liver will be a complex problem that requires more extensive future research.

It has been proved that antioxidants like HO‐1,^15^, ^58^ SOD2,^59^ GPX4^59^ play an important role in protecting from diabetes oxidative damage and keeping balance between health and disease.^59^ Apoptosis^60^ and autophagy^61^ are both regulated by oxidative stress. In the present study, we did not study the mechanisms by which T1D regulates autophagy and apoptosis.

## 5 CONCLUSIONS

Taken together our findings indicate that poorly controlled T1D does not cause severe hepatitis and NAFLD regardless of sex and age. Reduced lipids synthesis induced by insulin deficiency explained the declined levels of lipids in the liver. It is our assumption that changed apoptosis and autophagy might not only prevent the occurrence of acute liver injury meanwhile suppress liver self‐renewal, leading to hepatic dysfunction.

## CONFLICT OF INTEREST

All authors have declared that no conflict of interest exists.

## AUTHOR CONTRIBUTIONS

LC and SZJ conceived and designed the project. SZJ performed experiments, analysed data and wrote the draft of manuscript. KW performed experiments, analysed data and edited the manuscript. XQT and YQL conceived and performed experiments. YQ, CSL, LC analysed the data and edited the manuscript. LC is the guarantor of this work.

## References

[1] Petersen MC , Vatner DF , Shulman GI . Regulation of hepatic glucose metabolism in health and disease. Nat Rev Endocrinol. 2017;13:572‐587. 28731034 10.1038/nrendo.2017.80 PMC5777172

[2] Geisler CE , Renquist BJ . Hepatic lipid accumulation: cause and consequence of dysregulated glucoregulatory hormones. J Endocrinol. 2017;234:R1‐R21. 28428362 10.1530/JOE-16-0513

[3] Bhatt HB , Smith RJ . Fatty liver disease in diabetes mellitus. Hepatobiliary Surg Nutr. 2015;4:101‐108. 26005676 10.3978/j.issn.2304-3881.2015.01.03 PMC4405411

[4] Kim D , Touros A , Kim WR . Nonalcoholic fatty liver disease and metabolic syndrome. Clin Liver Dis. 2018;22:133‐140. 29128053 10.1016/j.cld.2017.08.010

[5] Epstein PN , Overbeek PA , Means AR . Calmodulin‐induced early‐onset diabetes in transgenic mice. Cell. 1989;58:1067‐1073. 2673540 10.1016/0092-8674(89)90505-9

[6] Carlson EC , Audette JL , Klevay LM , Nguyen H , Epstein PN . Ultrastructural and functional analyses of nephropathy in calmodulin‐induced diabetic transgenic mice. Anat Rec. 1997;247:9‐19. 8986297 10.1002/(SICI)1097-0185(199701)247:1<9::AID-AR2>3.0.CO;2-W

[7] Verges B . Lipid disorders in type 1 diabetes. Diabetes Metab. 2009;35:353‐360. 19733492 10.1016/j.diabet.2009.04.004

[8] Xu Z , Tong Q , Zhang Z , et al. Inhibition of HDAC3 prevents diabetic cardiomyopathy in OVE26 mice via epigenetic regulation of DUSP5‐ERK1/2 pathway. Clin Sci (Lond). 2017;131:1841‐1857. 28533215 10.1042/CS20170064 PMC5737625

[9] Zhang J , Xu Z , Gu J , et al. HDAC3 inhibition in diabetic mice may activate Nrf2 preventing diabetes‐induced liver damage and FGF21 synthesis and secretion leading to aortic protection. Am J Physiol Endocrinol Metab. 2018;315(2):E150‐E162. 29634312 10.1152/ajpendo.00465.2017

[10] Qian L‐B , Jiang S‐Z , Tang X‐Q , et al. Exacerbation of diabetic cardiac hypertrophy in OVE26 mice by angiotensin II is associated with JNK/c‐Jun/miR‐221‐mediated autophagy inhibition. Oncotarget. 2017;8:106661‐106671. 29290979 10.18632/oncotarget.21302 PMC5739764

[11] Yang LU , Brozovic S , Xu J , et al. Inflammatory gene expression in OVE26 diabetic kidney during the development of nephropathy. Nephron Exp Nephrol. 2011;119:e8‐20. 21606656 10.1159/000324407

[12] Zhang Q , Li Y , Liang T , et al. ER stress and autophagy dysfunction contribute to fatty liver in diabetic mice. Int J Biol Sci. 2015;11:559‐568. 25892963 10.7150/ijbs.10690 PMC4400387

[13] Cai LU , Wang Y , Zhou G , et al. Attenuation by metallothionein of early cardiac cell death via suppression of mitochondrial oxidative stress results in a prevention of diabetic cardiomyopathy. J Am Coll Cardiol. 2006;48:1688‐1697. 17045908 10.1016/j.jacc.2006.07.022

[14] Wang Y , Feng W , Xue W , et al. Inactivation of GSK‐3beta by metallothionein prevents diabetes‐related changes in cardiac energy metabolism, inflammation, nitrosative damage, and remodeling. Diabetes. 2009;58:1391‐1402. 19324938 10.2337/db08-1697 PMC2682666

[15] Gu J , Cheng Y , Wu H , et al. Metallothionein is downstream of Nrf2 and partially mediates sulforaphane prevention of diabetic cardiomyopathy. Diabetes. 2017;66:529‐542. 27903744 10.2337/db15-1274 PMC5248986

[16] Mehlem A , Hagberg CE , Muhl L , Eriksson U , Falkevall A . Imaging of neutral lipids by oil red O for analyzing the metabolic status in health and disease. Nat Protoc. 2013;8:1149‐1154. 23702831 10.1038/nprot.2013.055

[17] Lo S , Russell JC , Taylor AW . Determination of glycogen in small tissue samples. J Appl Physiol. 1970;28:234‐236. 5413312 10.1152/jappl.1970.28.2.234

[18] Becard D , Hainault I , Azzout‐Marniche D , Bertry‐Coussot L , Ferre P , Foufelle F . Adenovirus‐mediated overexpression of sterol regulatory element binding protein‐1c mimics insulin effects on hepatic gene expression and glucose homeostasis in diabetic mice. Diabetes. 2001;50:2425‐2430. 11679417 10.2337/diabetes.50.11.2425

[19] Yang L . Adriamycin nephrotoxicity is reduced by metallothionein over‐expression and kidney gene expression is modified by diabetes in the OVE26 diabetic model. Electronic Theses and Dissertations. 2010; Paper 1618.

[20] Kander MC , Cui Y , Liu Z . Gender difference in oxidative stress: a new look at the mechanisms for cardiovascular diseases. J Cell Mol Med. 2017;21:1024‐1032. 27957792 10.1111/jcmm.13038 PMC5387169

[21] Kapoor E , Collazo‐Clavell ML , Faubion SS . Weight gain in women at midlife: a concise review of the pathophysiology and strategies for management. Mayo Clin Proc. 2017;92:1552‐1558. 28982486 10.1016/j.mayocp.2017.08.004

[22] Leeners B , Geary N , Tobler PN , Asarian L . Ovarian hormones and obesity. Human Reproduction Update. 2017;23:300‐321. 28333235 10.1093/humupd/dmw045 PMC5850121

[23] Wellons MF , Matthews JJ , Kim C . Ovarian aging in women with diabetes: an overview. Maturitas. 2017;96:109‐113. 28041589 10.1016/j.maturitas.2016.11.019 PMC5268844

[24] Codner E . Estrogen and type 1 diabetes mellitus. Pediatr Endocrinol Rev. 2008;6:228‐234. 19202509

[25] Targher G , Bertolini L , Padovani R , et al. Prevalence of non‐alcoholic fatty liver disease and its association with cardiovascular disease in patients with type 1 diabetes. J Hepatol. 2010;53:713‐718. 20619918 10.1016/j.jhep.2010.04.030

[26] Lăpădat AM , Jianu IR , Ungureanu BS , et al. Non‐invasive imaging techniques in assessing non‐alcoholic fatty liver disease: a current status of available methods. J Med Life. 2017;10:19‐26. 28255371 PMC5304366

[27] Kummer S , Klee D , Kircheis G , et al. Screening for non‐alcoholic fatty liver disease in children and adolescents with type 1 diabetes mellitus: a cross‐sectional analysis. Eur J Pediatr. 2017;176:529‐536. 28213828 10.1007/s00431-017-2876-1

[28] Petit JM , Pedro L , Guiu B , et al. Type 1 diabetes is not associated with an increased prevalence of hepatic steatosis. Diabet Med. 2015;32:1648‐1651. 25981893 10.1111/dme.12805

[29] Perseghin G , Lattuada G , De Cobelli F , et al. Reduced intrahepatic fat content is associated with increased whole‐body lipid oxidation in patients with type 1 diabetes. Diabetologia. 2005;48:2615‐2621. 16261312 10.1007/s00125-005-0014-5

[30] Cusi K , Sanyal AJ , Zhang S , et al. Non‐alcoholic fatty liver disease (NAFLD) prevalence and its metabolic associations in patients with type 1 diabetes and type 2 diabetes. Diabetes Obes Metab. 2017;19(11):1630‐1634. 28417532 10.1111/dom.12973

[31] Regnell SE , Peterson P , Trinh L , et al. Magnetic resonance imaging reveals altered distribution of hepatic fat in children with type 1 diabetes compared to controls. Metabolism. 2015;64:872‐878. 25982699 10.1016/j.metabol.2015.04.002

[32] Bunner AE , Chandrasekera PC , Barnard ND . Knockout mouse models of insulin signaling: relevance past and future. World J Diabetes. 2014;5:146‐159. 24748928 10.4239/wjd.v5.i2.146 PMC3990311

[33] Titchenell PM , Lazar MA , Birnbaum MJ . Unraveling the regulation of hepatic metabolism by insulin. Trends Endocrinol Metab. 2017;28:497‐505. 28416361 10.1016/j.tem.2017.03.003 PMC5477655

[34] Mittendorfer B , Klein S . Absence of leptin triggers type 1 diabetes. Nat Med. 2014;20:705‐706. 24999939 10.1038/nm.3629

[35] American DA . Diagnosis and classification of diabetes mellitus. Diabetes Care. 2012;35(Suppl 1):S64‐71. 22187472 10.2337/dc12-s064 PMC3632174

[36] Purnell JQ , Zinman B , Brunzell JD , et al. The effect of excess weight gain with intensive diabetes mellitus treatment on cardiovascular disease risk factors and atherosclerosis in type 1 diabetes mellitus: results from the Diabetes Control and Complications Trial/Epidemiology of Diabetes Interventions and Complications Study (DCCT/EDIC) study. Circulation. 2013;127:180‐187. 23212717 10.1161/CIRCULATIONAHA.111.077487 PMC3819101

[37] Cohen JC , Horton JD , Hobbs HH . Human fatty liver disease: old questions and new insights. Science. 2011;332:1519‐1523. 21700865 10.1126/science.1204265 PMC3229276

[38] Pawlak M , Lefebvre P , Staels B . Molecular mechanism of PPARalpha action and its impact on lipid metabolism, inflammation and fibrosis in non‐alcoholic fatty liver disease. J Hepatol. 2015;62:720‐733. 25450203 10.1016/j.jhep.2014.10.039

[39] Miquilena‐Colina ME , Lima‐Cabello E , Sanchez‐Campos S , et al. Hepatic fatty acid translocase CD36 upregulation is associated with insulin resistance, hyperinsulinaemia and increased steatosis in non‐alcoholic steatohepatitis and chronic hepatitis C. Gut. 2011;60:1394‐1402. 21270117 10.1136/gut.2010.222844

[40] Wilson CG , Tran JL , Erion DM , Vera NB , Febbraio M , Weiss EJ . Hepatocyte‐specific disruption of CD36 attenuates fatty liver and improves insulin sensitivity in HFD‐Fed Mice. Endocrinology. 2016;157:570‐585. 26650570 10.1210/en.2015-1866 PMC4733118

[41] Chatila R , West AB . Hepatomegaly and abnormal liver tests due to glycogenosis in adults with diabetes. Medicine (Baltimore). 1996;75:327‐333. 8982149 10.1097/00005792-199611000-00003

[42] Bischof MG , Krssak M , Krebs M , et al. Effects of short‐term improvement of insulin treatment and glycemia on hepatic glycogen metabolism in type 1 diabetes. Diabetes. 2001;50:392‐398. 11272152 10.2337/diabetes.50.2.392

[43] Goran MI , Gower BA . Longitudinal study on pubertal insulin resistance. Diabetes. 2001;50:2444‐2450. 11679420 10.2337/diabetes.50.11.2444

[44] Al‐Hussaini AA , Sulaiman NM , AlZahrani MD , Alenizi AS , Khan M . Prevalence of hepatopathy in type 1 diabetic children. BMC Pediatr. 2012;12:160. 23039762 10.1186/1471-2431-12-160 PMC3506494

[45] Stadler M , Bollow E , Fritsch M , et al. Prevalence of elevated liver enzymes in adults with type 1 diabetes: a multicentre analysis of the German/Austrian DPV database. Diabetes Obes Metab. 2017;19:1171‐1178. 28256088 10.1111/dom.12929

[46] Elkabbany ZA , Elbarbary NS , Ismail EA , et al. Transient elastography as a noninvasive assessment tool for hepatopathies of different etiology in pediatric type 1 diabetes mellitus. J Diabetes Complications. 2017;31:186‐194. 27742550 10.1016/j.jdiacomp.2016.09.009

[47] Harman DJ , Kaye PV , Harris R , Suzuki A , Gazis A , Aithal GP . Prevalence and natural history of histologically proven chronic liver disease in a longitudinal cohort of patients with type 1 diabetes. Hepatology. 2014;60:158‐168. 24585431 10.1002/hep.27098

[48] Arroyo DS , Gaviglio EA , Peralta Ramos JM , Bussi C , Rodriguez‐Galan MC , Iribarren P . Autophagy in inflammation, infection, neurodegeneration and cancer. Int Immunopharmacol. 2014;18:55‐65. 24262302 10.1016/j.intimp.2013.11.001 PMC3903161

[49] Shintani T , Klionsky DJ . Autophagy in health and disease: a double‐edged sword. Science. 2004;306:990‐995. 15528435 10.1126/science.1099993 PMC1705980

[50] Ni H‐M , Woolbright BL , Williams J , et al. Nrf2 promotes the development of fibrosis and tumorigenesis in mice with defective hepatic autophagy. J Hepatol. 2014;61:617‐625. 24815875 10.1016/j.jhep.2014.04.043 PMC4143992

[51] Vanhorebeek I , Gunst J , Derde S , et al. Insufficient activation of autophagy allows cellular damage to accumulate in critically ill patients. J Clin Endocrinol Metab. 2011;96:E633‐E645. 21270330 10.1210/jc.2010-2563

[52] Thiessen SE , Derese I , Derde S , et al. The role of autophagy in critical illness‐induced liver damage. Sci Rep. 2017;7:14150. 29074879 10.1038/s41598-017-14405-w PMC5658339

[53] Singh R , Kaushik S , Wang Y , et al. Autophagy regulates lipid metabolism. Nature. 2009;458:1131‐1135. 19339967 10.1038/nature07976 PMC2676208

[54] Westhoff M‐A , Brühl O , Nonnenmacher L , Karpel‐Massler G , Debatin K‐M . Killing me softly–future challenges in apoptosis research. Int J Mol Sci. 2014;15:3746‐3767. 24595238 10.3390/ijms15033746 PMC3975365

[55] Chowdhury I , Tharakan B , Bhat GK . Current concepts in apoptosis: the physiological suicide program revisited. Cell Mol Biol Lett. 2006;11:506‐525. 16977376 10.2478/s11658-006-0041-3 PMC6275981

[56] Schattenberg JM , Galle PR , Schuchmann M . Apoptosis in liver disease. Liver Int. 2006;26:904‐911. 16953829 10.1111/j.1478-3231.2006.01324.x

[57] Luedde T , Kaplowitz N , Schwabe RF . Cell death and cell death responses in liver disease: mechanisms and clinical relevance. Gastroenterology. 2014;147:765‐783.e4. 25046161 10.1053/j.gastro.2014.07.018 PMC4531834

[58] Kah J , Volz T , Lütgehetmann M , et al. Haem oxygenase‐1 polymorphisms can affect HCV replication and treatment responses with different efficacy in humanized mice. Liver Int. 2017;37:1128‐1137. 27992676 10.1111/liv.13347

[59] Li S , Tan H‐Y , Wang N , et al. The role of oxidative stress and antioxidants in liver diseases. Int J Mol Sci. 2015;16:26087‐26124. 26540040 10.3390/ijms161125942 PMC4661801

[60] Curtin JF , Donovan M , Cotter TG . Regulation and measurement of oxidative stress in apoptosis. J Immunol Methods. 2002;265:49‐72. 12072178 10.1016/s0022-1759(02)00070-4

[61] Filomeni G , De Zio D , Cecconi F . Oxidative stress and autophagy: the clash between damage and metabolic needs. Cell Death Differ. 2015;22:377‐388. 25257172 10.1038/cdd.2014.150 PMC4326572

