# Supplementary figures and images for: Hepatic functional and pathological changes of type 1 diabetic mice in growing and maturation time
Source: J Cell Mol Med. 2019 Jun 20;23(8):5794–807. doi: 10.1111/jcmm.14504 (PMC6652934; doi:10.1111/jcmm.14504)

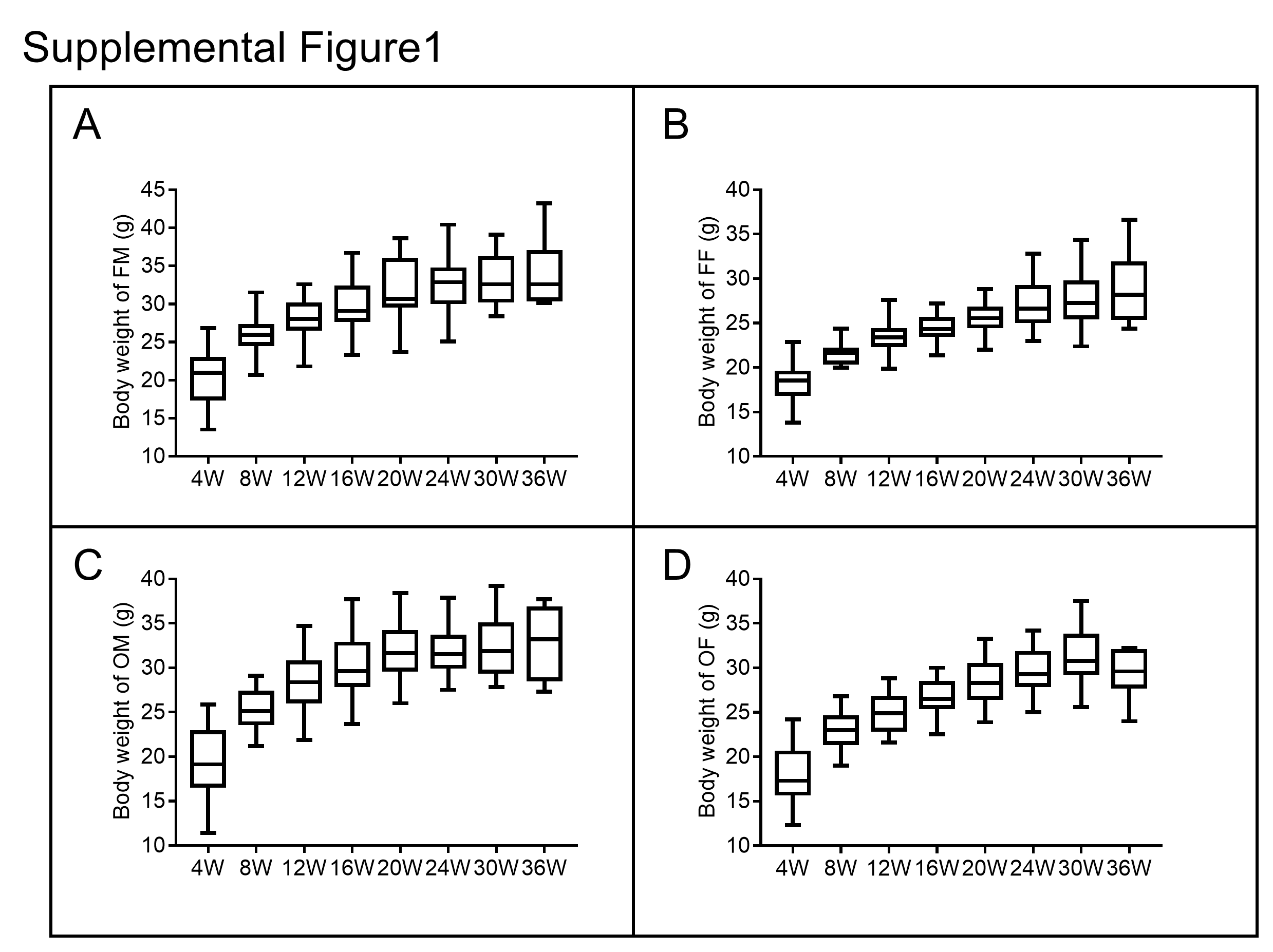

Supplement: Supplementary file 1 [file JCMM-23-5794-s001.tif]

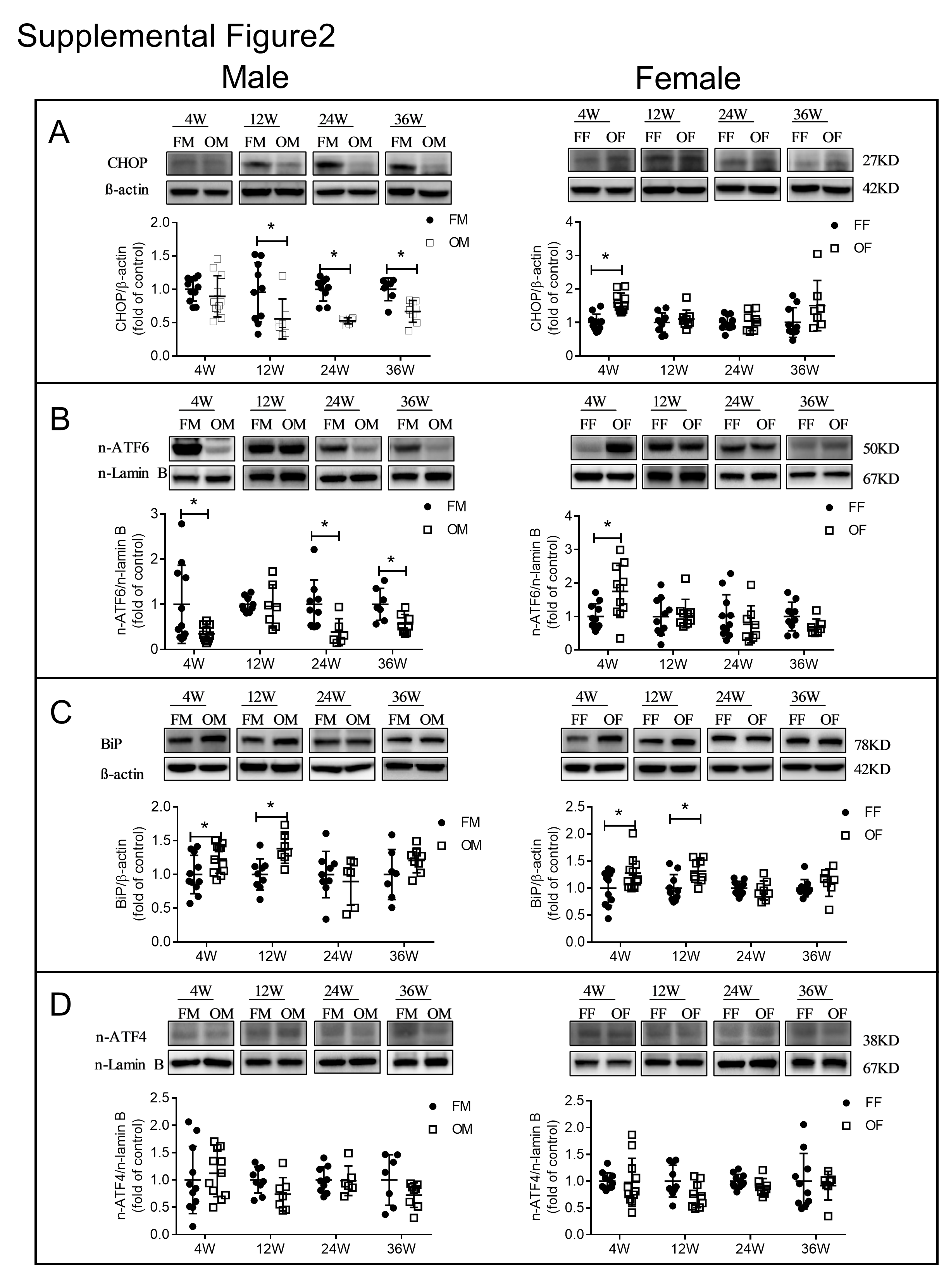

Supplement: Supplementary file 2 [file JCMM-23-5794-s002.tif]
