# Supplementary material for: Hepatic functional and pathological changes of type 1 diabetic mice in growing and maturation time
Source: J Cell Mol Med. 2019 Jun 20;23(8):5794–807. doi: 10.1111/jcmm.14504 (PMC6652934; doi:10.1111/jcmm.14504)
Supplement: Supplementary file 3 [file JCMM-23-5794-s003.docx]

**1. Supplemental Tables**

**1.1. Supplemental Table 1: List of antibody for western blot and immunofluorescence**

| **Number** | **Name** | **Company** | **Dilution** |
| --- | --- | --- | --- |
| **For western blot** | | |  |
| **1** | Superoxide dismutase 2(SOD2) sc-133134 | Santa Cruz Biotechnology | 1;1000 |
| **2** | Lamin B1 sc-374015 | Santa Cruz Biotechnology | 1;1000 |
| **3** | β-actin sc-47778 | Santa Cruz Biotechnology | 1;3000 |
| **4** | Tumor necrosis factor alpha (TNF-α) sc-52746 | Santa Cruz Biotechnology | 1;1000 |
| **5** | SQSTM1/p62 5114 | Cell Signaling | 1;1000 |
| **6** | Binding immunoglobulin protein (BiP) 3177 | Cell Signaling | 1;1000 |
| **7** | Caspase-3 9662 | Cell Signaling | 1;1000 |
| **8** | CCAAT-enhancer-binding protein homologous protein (CHOP)  2895 | Cell Signaling | 1;1000 |
| **9** | Apoptosis-inducing factor (AIF) 5318 | Cell Signaling |  |
| **10** | Activating transcription factor 4( ATF4) ab1371 | Abcam | 1;1000 |
| **11** | Fatty Acid Synthase(FAS) ab22759 | Abcam | 1;1000 |
| **12** | Glutathione Peroxidase 4(GPX4) ab125066 | Abcam | 1;1000 |
| **13** | Heme Oxygenase 1(HO-1) ab13248 | Abcam | 1;1000 |
| **14** | SREBP1 ab28481 | Abcam | 1;1000 |
| **15** | LC3B | Novus Biologicals | 1;1000 |
| **16** | peroxisome proliferator activated receptor (PPAR) alpha PA1-822A | Thermal Fisher | 1;1000 |
| **17** | Activating transcription factor 6(ATF6) 70B1413.1 | Novus | 1;1000 |
| **18** | 3-nitrotyrosine (3-NT) ab5411 | Millipore | 1;1000 |
| **19** | 4-Hydroxy-2-nonenal (HNE) HNE11-S | Alpha Diagnostic Int. | 1;1000 |
| **20** | Goat F(ab) Anti-Mouse IgG H&L ab6668 | Abcam | 1;3000 |
| **21** | HRP-conjugated IgG M:7076s R:7074s | Cell Signaling | 1;3000 |
| **For immunofluorescence** | | |  |
| **24** | Phalloidin | Thermal Fisher | 1;50 |

**1.2. Supplemental Table 2: List of primer for quantitative real-time PCR (qRT-PCR)**

| **Number** | **Name** | **Company** | **Cat No** |
| --- | --- | --- | --- |
| **1** | stearoyl-Coenzyme A desaturase 1（SCD1） | Thermal Fisher | Mm00772290_m1 |
| **2** | CD36 antigen (CD36) | Thermal Fisher | Mm01135198_m1 |
